# Logical error rate in the Pauli twirling approximation

**DOI:** 10.1038/srep14670

**Published:** 2015-09-30

**Authors:** Amara Katabarwa, Michael R. Geller

**Affiliations:** 1 Department of Physics and Astronomy, University of Georgia, Athens, Georgia 30602, USA

## Abstract

The performance of error correction protocols are necessary for understanding the operation of potential quantum computers, but this requires physical error models that can be simulated efficiently with classical computers. The Gottesmann-Knill theorem guarantees a class of such error models. Of these, one of the simplest is the Pauli twirling approximation (PTA), which is obtained by twirling an arbitrary completely positive error channel over the Pauli basis, resulting in a Pauli channel. In this work, we test the PTA’s accuracy at predicting the logical error rate by simulating the 5-qubit code using a 9-qubit circuit with realistic decoherence and unitary gate errors. We find evidence for good agreement with exact simulation, with the PTA *overestimating* the logical error rate by a factor of 2 to 3. Our results suggest that the PTA is a reliable predictor of the logical error rate, at least for low-distance codes.

Feynman introduced the idea of a universal quantum simulator^1^, noting that a classical Turing machine would require a time exponential in the number of particles to simulate quantum phenomena, while his proposed simulator made from quantum components would avoid such a scaling. A surge of interest grew around the nascent field of quantum information theory when Shor discovered his now famous algorithm^2^, which can provably factor numbers in polynomial time, in contrast to a classical machine which is believed to scale exponentially with the number of bits of the input. However, it was clear from the very beginning that the great power of quantum computing—using quantum superpositions and entanglement—also presented the greatest challenge to its realization; namely, the incredible delicacy of quantum states in the presence of unwanted environmental interactions.

The first major step to protect the delicacy was taken by Shor when he proposed a quantum circuit that could correct for any single-qubit error by encoding a logical qubit into 9 physical qubits^3^. Shor’s 9-qubit code and it’s generalizations work perfectly if we make the unphysical assumption that all syndrome measurements are error-free. It is therefore necessary to understand the effect of decoherence and unitary gate errors on a complete fault-tolerant circuit. Unfortunately, the direct approach to this problem, namely a full Hilbert space simulation of the quantum circuit in the presence of errors and noise, is impractical because of the exponential relationship between amount of memory and time needed to simulate quantum circuits and the number of qubits. A way around this problem is to rely on the Gottesmann-Knill theorem, which shows that any circuit in which we prepare initial states in the computational basis, use only gates from the normalizer of the Pauli group (in this case the Clifford group), and measure operators from the Pauli group, can be efficiently simulated on a classical computer^4^. We are thus provided with a class of efficient error models that includes the Pauli and Clifford channels.

Simulation of a noisy quantum circuit is accomplished by performing each ideal operation followed by an error (a gate from Pauli or Clifford group) with some probability. It is then necessary to construct an error channel such that one approximates the true noise process as accurately as possible (with respect to some measure), and ideally with the additional property that the approximate channel upper-bounds the actual error. The first steps in this direction were taken by Magesan *et al.*^5^ and Gutiérrez *et al.*^6^. These investigations considered a single qubit density matrix and not a quantum error correcting circuit. Geller and Zhou^7^ took a different approach and asked how well the Pauli twirling approximation (PTA), obtained by twirling the exact error channel over the Pauli basis, performed on a 4-qubit Bell-state preservation circuit, where an analog of the logical error rate can be defined. Despite its simplicity, the PTA was found to work surprising well over a large range of physical error rates, but did not always upper bound the exact error. A second test of the PTA was carried out by Tomita and Svore^8^, where the logical error rate was calculated for the distance-3 surface code. Although the PTA test was not the main focus of their work, these authors found excellent agreement for logical *σ*^*z*^ errors, but that the PTA overestimated the logical *σ*^*x*^ error by a factor of 5 to 10, depending on the qubit *T*_1_ time. The results of Tomita and Svore^8^, and the desire to extend the work of ref. 7 to a test of the PTA on an actual logical error rate calculation, motivated the work reported here. In addition, two other related investigations have recently appeared: Puzzuoli *et al.*^9^ discussed the construction of efficient (Pauli and Clifford) error channels obtained by minimizing the diamond norm subject to the constraint that the approximate channel always upper bounds the error (an *honest* representation in the terminology of refs 5,9) and tested their accuracy when applied to error-correcting circuits. Gutiérrez and Brown^10^ focused on Clifford channels and computed error thresholds for the Steane^1^,^3^,^7^ code. The results of refs 7,8,10, together with the results reported below, suggest that the PTA is a reliable (and honest) predictor of the logical error rate, at least for low-distance codes.

## Methods and Results

Consider the time evolution of a density matrix *ρ* represented by some superoperator Λ; then we have^11^
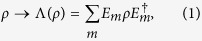
where the *E*_*m*_ are *N* × *N* Kraus matrices. Next consider a finite set of operations 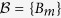 with *m* = 1, …, *K. Twirling*^12^,^13^,^14^,^15^ the channel to obtain a new channel 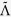 is to perform the operation
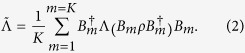


To arrive at the PTA, we simply consider the set 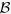 to be the n-qubit Pauli basis 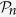, defined as consisting of all possible tensor products
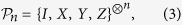
giving a total of 4^*n*^ distinct elements. Performing the PTA gives 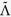 that is always diagonal in the Pauli basis, namely
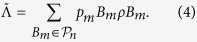


If Λ is trace-preserving then 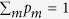, otherwise 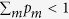. A detailed application of the PTA to single qubit decoherence models is carried out in ref. 7.

In this work we apply the PTA to the calculation of the logical error rate for the 5-qubit code. The 5-qubit code is the smallest quantum error correcting code that can encode a logical qubit, and detect and correct a single one-qubit error. It is a distance 3 quantum error correcting circuit, meaning that with one error correcting cycle, 3 is the lowest number of single qubit errors that cannot be detected. This code can be implemented by measuring the stabilizers
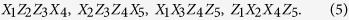


Note that by starting with the first stabilizer one can arrive at the other three by a cyclic permutation of the qubits. The logical 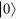 and logical 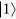 states for this code are
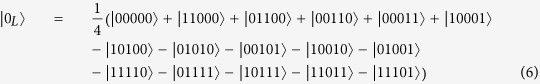
and
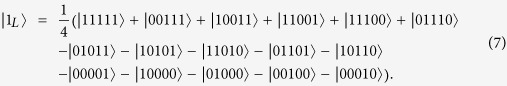


**Figure 1:**
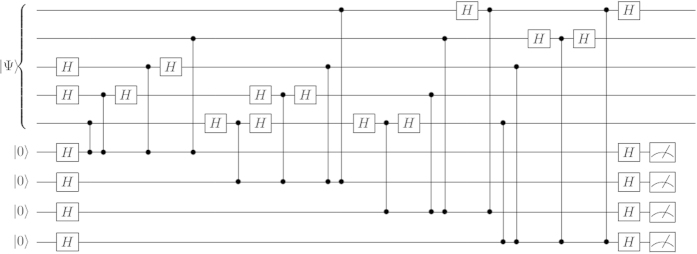
Stabilizer measurement circuit for the 5-qubit code written in terms of CZ gates (vertical lines with dots). A *cycle* is moving through this circuit once and performing the measurement step.

A logical state is prepared in the computational basis using the first five (data) qubits, as shown in Fig. 1. The next four qubits are used as ancilla qubits to measure the four stabilizers after which four measurement outcomes (*x*_1_, *x*_2_, *x*_3_, *x*_4_) are obtained. There are 16 possible measurement outcomes which are in a one to one correspondence with the 16 possible errors that might occur [counting the outcome (0, 0, 0, 0) as a trivial error]. In Table 1 we list all possible measurement outcomes and the corresponding single qubit errors. We call the implementation of the circuit and performing the measurement step a *cycle*, which is shown in Fig. 1. If one goes through a cycle and a single error occurs on any of the first 5 qubits, this might be reflected in the measurement result and thus detected. But instead suppose that no errors occur on the data qubits but right before the measurement step a bit-flip error occurs on the first syndrome qubit, giving the measurement outcome of (1, 0, 0, 0). An incorrect interpretation of the result would be to conclude that one of the 16 possible errors on the data qubits has occurred, whereas in fact the fault lies with a syndrome qubit. We therefore require a protocol that is tolerant to a single syndrome-qubit (or readout) error. To this end, we note that for errors uncorrelated in time, it is likely that after readout and re-initialization that the syndrome qubit will return to its original “faithful” state at the end of the next cycle. The procedure followed in our simulations is therefore the following:

1. With the initial 9-qubit density matrix (representing data and syndrome qubits) perform the stabilizer measurements and the measurement step to complete one cycle. Record the measurement outcome.
2. For the next cycle, re-initialize the syndrome qubits but use the 5-qubit density matrix from the end of the last cycle.
3. Repeat step 2 until the same measurement outcome is obtained three times in succession. Call this event the completion of a *trial*.

After observing the same measurement outcome for three cycles in a row, we calculate
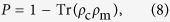
where *ρ*_*c*_ is the data-qubit density matrix obtained at the end of the final cycle and *ρ*_*m*_ is the data-qubit density matrix predicted by the stable measurement outcome. We then define the logical error rate as
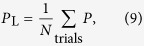
where *N* is the number of trials. The value of N is chosen to make the sampling errors much smaller than the differences between the exact and PTA logical error rates we are interested in. Defining the logical error rate this way allows us to calculate an error rate that could be measured experimentally.

Our work here is done with surface code in mind where for example one deals with single syndrome qubits and the robustness of a syndrome measurement is achieved by comparing measurement results from a number of measurement cycles.

For the exact calculations of the average logical error rate *P*_L_, decoherence was included by using Kraus matrices for amplitude damping and pure dephasing as defined in ref. 7. The gates are assumed to act instantaneously and the non-unitary evolution is implemented using the operator sum representation between the action of the gates for a time of 25 × 10^−9^ s. Unitary gate errors are introduced by using the non-ideal CZ gate^7^
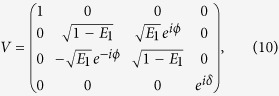
whereas the Hadamards are taken to be ideal. The form (10) reflects actual errors in a CZ gate implemented with superconducting qubits (neglecting leakage)^16^. There are three parameters in (10) that can be changed, namely, *E*_1_, *ϕ* and *δ*. In the simulations, we choose *ϕ* = 0 and distribute the total intrinsic gate error *E* equally between *E*_1_ and *δ*. By *intrinsic* or unitary gate error we mean the gate error in the absence of decoherence. The PTA applied to (10) yields 16 two-qubit Pauli error operators with probabilities given in ref. 7. To obtain standard errors on the order 10^−3^ or smaller, about 20 trials were required, which took several days of runtime to complete. The PTA calculations of *P*_L_ were done using classical Monte Carlo, which introduces larger sampling errors. A total of 10000 trials were performed to get sampling errors down to around 10^−3^.

**Figure 2:**
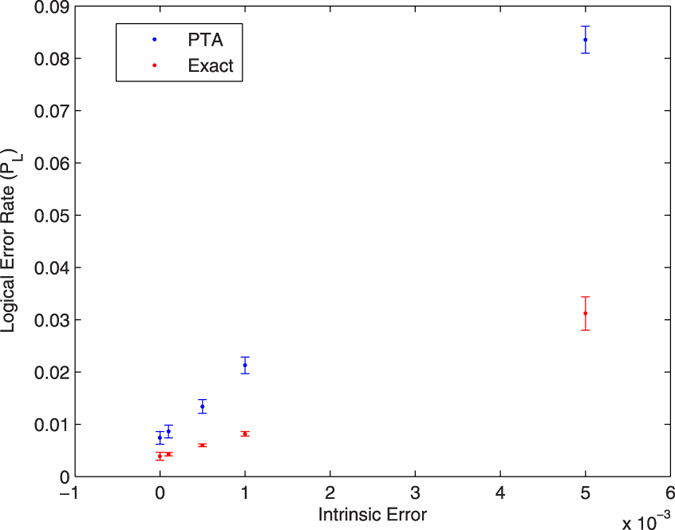
Logical error rate for the |0〉_*L*_ state with *T*_1_ = *T*_2_ 100 *μs*.

**Figure 3:**
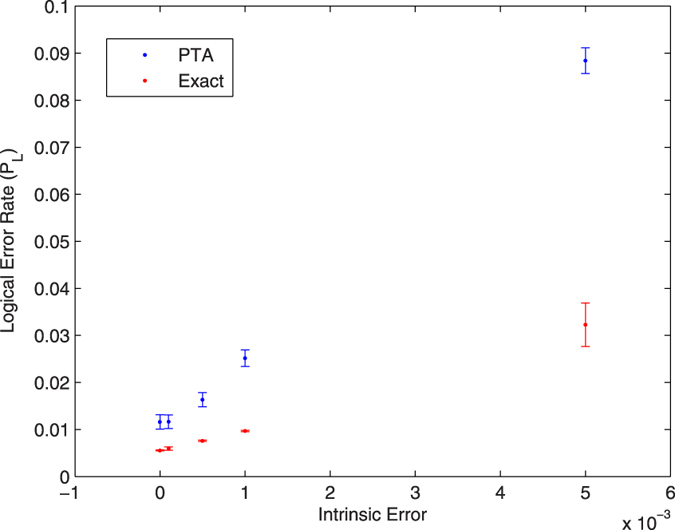
Logical error rate for the |0〉_*L*_ state with *T*_1_ = *T*_2_ 70 *μs*.

**Figure 4:**
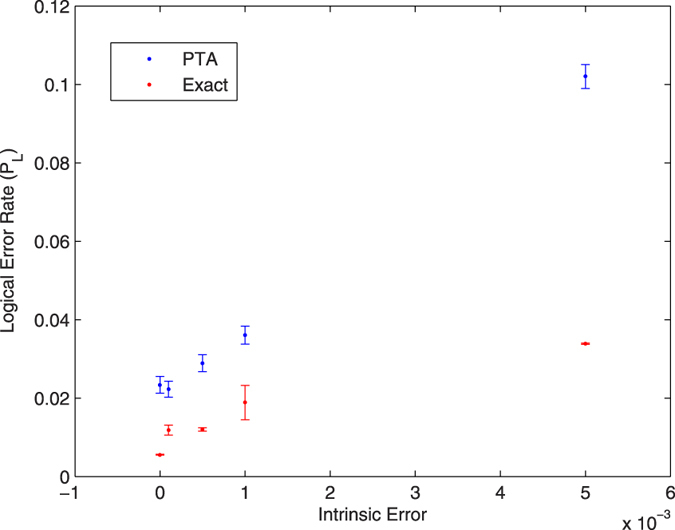
Logical error rate for the |0〉_*L*_ state with *T*_1_ = *T*_2_ 40 *μs*.

**Figure 5:**
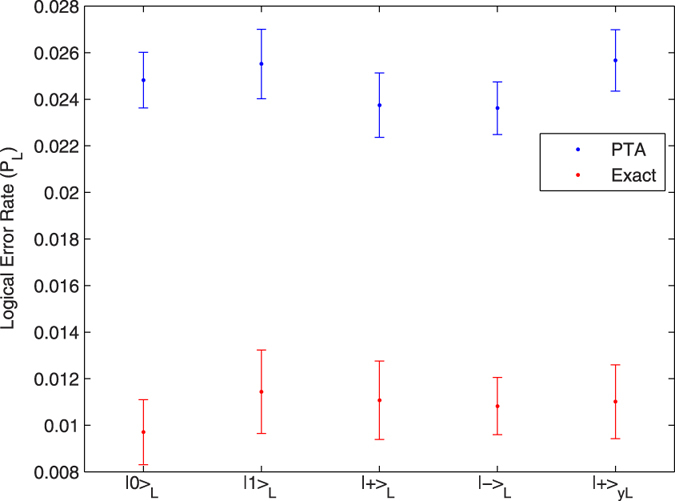
Logical error rate for different states on the Bloch sphere with *T*_1_ = *T*_2_ 70 *μs*. and 10^−3^ intrinsic error.

Figures 2,3 and 4 give the logical error rate *P*_L_ versus intrinsic error for three values of *T*_1_, with *T*_2_ = *T*_1_. We find in these cases that the PTA overestimates the logical error rate by about a factor of 2 to 3. In Fig. 5, we fix the total intrinsic error to *E* = 10^−3^ and test the PTA for five different states on the logical Bloch sphere: the eigenstates of *σ*^*z*^, *σ*^*x*^, and the +1 eigenstate of *σ*^*y*^.

## Conclusions

We have studied the PTA logical error rate compared to an exact calculation that includes both decoherence (amplitude damping and pure dephasing) and unitary gate errors. The tests reported here include 20 different settings—physical error rates and/or initial logical states—with the PTA *overestimating* the logical error rate by a factor of 1.9 to 3.1, with a mean ratio of 2.35. In the language of Megasan *et al.*^5^, we find that the PTA is always honest (the ratio is >1) for the parameter regimes considered. We find no significant difference between PTA’s performance for bit-flip and phase-flip errors, which would be reflected in Fig. 5, in contrast to the results of Tomita and Svore^8^. The explanation for the difference between the results is currently not known. There are a number of significant differences between the two arenas in which PTA’s performance is measured that could be the explanation: the fact that in surface code we have a two dimensional structure which allows for more interactions between the qubits,the fact that one is topological and the other is not, or it could be as simple as the work in ref. 8 studied a considerably larger system. This warrants future study. We also find that, as expected, the PTA is less accurate for unitary errors, in agreement with refs 7,8.

## Additional Information

**How to cite this article**: Katabarwa, A. and Geller, M. R. Logical error rate in the Pauli twirling approximation. *Sci. Rep.* **5**, 14670; doi: 10.1038/srep14670 (2015).

## Figures and Tables

**Table 1:** Syndrome measurement outcomes and their corresponding predicted single-qubit errors.

measurement result	single-qubit error
0000	I
0001	*Z*_1_
0010	*X*_3_
0011	*Z*_0_
0100	*X*_0_
0101	*X*_2_
0110	*Z*_4_
0111	*Y*_0_
1000	*Z*_2_
1001	*X*_4_
1010	*X*_1_
1011	*Y*_1_
1100	*Z*_3_
1101	*Y*_2_
1110	*Y*_3_
1111	*Y*_4_
